# Characterization of the role of TMEM45A in cancer cell sensitivity to cisplatin

**DOI:** 10.1038/s41419-019-2088-x

**Published:** 2019-12-04

**Authors:** Kathleen Schmit, Jia-Wei Chen, Sophie Ayama-Canden, Maude Fransolet, Laure Finet, Catherine Demazy, Lionel D’Hondt, Carlos Graux, Carine Michiels

**Affiliations:** 1 URBC-NARILIS, University of Namur, Namur, Belgium; 2 Université Catholique de Louvain, CHU UCL Namur, Biobank, Yvoir, Belgium

**Keywords:** Cancer therapy, Head and neck cancer, Tumour biomarkers, Apoptosis

## Abstract

TMEM45A is a transmembrane protein involved in tumor progression and cancer resistance to chemotherapeutic agents in hypoxic condition. It is correlated to a low breast cancer patient overall survival. However, little is known about this protein, in particular the mechanisms by which TMEM45A modulates cancer cell chemosensitivity. In this work, the messenger RNA expression of TMEM45A was assessed in head and neck squamous cell carcinoma (HNSCC) and renal cell carcinoma (RCC) biopsies. TMEM45A was upregulated in patients diagnosed for head and neck or renal cancer. Then, the implication of this protein in cisplatin sensitivity was explored in SQD9 and RCC4 + pVHL cells. TMEM45A inactivation decreased cell proliferation and modulated cell responses to cisplatin. Indeed, TMEM45A inactivation increased the sensitivity of SQD9 cells to cisplatin, whereas it rendered RCC4 + pVHL cells resistant to this anticancer agent. Through RNA-sequencing analysis, we identified several deregulated pathways that indicated that the impact on cisplatin sensitivity may be associated to the inhibition of DNA damage repair and to UPR pathway activation. This study demonstrated, for the first time, an anti or a pro-apoptotic role of this protein depending on the cancer type and highlighted the role of TMEM45A in modulating patient responses to treatment.

## Introduction

Renal cell carcinoma (RCC) and head and neck squamous cell carcinoma (HNSCC) constitute two of the ten most common cancers^1,2^. International Agency for Research on Cancer recorded >350,000 new cases of HNSCC worldwide in 2018, with 25% of oral cancer being HPV-positive^3^. The current standard treatments for this disease are surgery, radiotherapy, and chemotherapy. The four most extensively used agents are methotrexate, cisplatin, 5-fluorouracil (5-FU), and bleomycin. Over the years, significant advances have been made in combined modality therapies but the majority of patients who present advanced disease relapse still develop metastases and only qualify for palliative treatment^4^.

Human RCCs are classified in subtypes with the most common being clear cell RCC (ccRCC), which represents 75–80% of the group^5^. von Hippel-Lindau (VHL) tumor suppressor gene is the most frequently mutated gene^6,7^. ccRCC can be treated by surgical excision or with ablative therapies, but also with systemic therapies. The most used drugs are angiogenesis targeting agents, including sorafenib, sunitinib, and bevacizumab, which target vascular endothelial growth factor (VEGF) or its receptor (VEGFR). Despite all the therapeutic targeting agents discovered during the last decade, many patients are resistant to these treatments or acquire resistance over time^8,9^. Furthermore, due to the ability of the kidney to functionally compensate when partially damaged^10^, most patients are diagnosed with advanced or metastatic RCC.

Transmembrane protein family (TMEMs) is a family of proteins predicted to be components of various cell membranes, such as mitochondrial, endoplasmic reticulum, and golgi membranes. In many cancers, differential expression of TMEMs is observed and a large number of these proteins have been implicated in cancer development and drug resistance^11^.

TMEM45A is a transmembrane protein of 275 amino acids, predicted to have five or seven transmembrane domains and which is upregulated in hypoxic condition. This protein is highly expressed in the skin, is associated with trans-golgi apparatus and whose expression is correlated with epiderm keratinization^12–14^. TMEM45A has been proposed as a potential classifier of ccRCC^15^, but was also associated with chemoresistance in human breast cancer cells and in human hepatoma cells in hypoxic condition^16^. TMEM45A affects proliferation and invasion in human ovarian cancer cells and in human glioma cells^17,18^. In the cases of breast cancer and cervical lesions, higher expression level of TMEM45A has been correlated with lower patient overall survival, suggesting that TMEM45A could be a potential biomarker for aggressiveness^16,19^.

Since the expression of this protein has been associated with tumor aggressiveness, we studied the possible role of TMEM45A in cancer resistance to chemotherapies and the mechanism by which it acts. The effect of TMEM45A inactivation was investigated on responses to cisplatin in head and neck and renal cancer cells in normoxic and hypoxic conditions. Our data support an implication of TMEM45A in the sensitivity of cancer cells to cisplatin.

## Materials and methods

### Patient study

After written informed consent of patients (Biobank CHU-UCL Namur, Belgium), healthy tissues, which were histologically free from tumor and head and neck and renal cancer biopsies, were obtained from surgery. The Medical Ethical Committee of CHU-UCL Namur approved all described studies. After resection, the samples were stored at −80 °C and messenger RNA (mRNA) were extracted as described below. Primary tumor specimens and paired noncancerous tissues were obtained from 22 patients who were diagnosed with HNSCC. The ORL specimens analyzed in this study were obtained from patients of both gender with HNSCC, who were diagnosed between August 2010 and April 2018. The age of patients ranged from 43 to 77 years, with a median age of 57 years. Primary tumor specimens and paired noncancerous tissues were obtained from 25 patients who were diagnosed with RCC. The renal specimens analyzed in this study were obtained from patients of both gender with RCC, who were diagnosed between October 2011 and October 2015. The age of patients ranged from 43 to 91 years, with a median age of 67 years.

TCGA analysis of samples from human tumors and corresponding healthy tissues were performed for five cancer types: ESCA: esophageal carcinoma, HNSC: head and neck squamous carcinoma, KICH, kidney chromophobe, KIRC, kidney renal clear cell carcinoma, KIRP kidney renal papillary cell carcinoma (http://gepia.cancer-pku.cn/detail.php?gene = TMEM45A&clicktag = expdiy).

### Cell culture

Head and neck squamous cell carcinoma cell lines (SQD9 and Cal 27) were obtained from Pierre Sonveaux laboratory, Woluwe, Belgium and maintained in culture in 75 cm^2^ polystyrene flasks (Corning) in Minimum Essential Media (MEM) (Thermo Fisher Scientific) supplemented with 10% of heat inactivated fetal bovine serum (FBS) (Thermo Fisher Scientific). Renal cell carcinoma cell line RCC4 stably transfected with pcDNA3-VHL (RCC4 plus pVHL) was purchased from the European Collection of Authenticated Cell Cultures (Sigma-Aldrich) and maintained in culture in 75 cm^2^ polystyrene flasks (Corning) in Dulbecco’s Modified Eagle’s Medium (DMEM) (Thermo Fisher Scientific) supplemented with 2 mM of l-glutamine (Sigma-Aldrich), 10% of fetal bovine serum (FBS) (Thermo Fisher Scientific), and geneticin (0.5 mg/mL) (Thermo Fisher Scientific). Cells were incubated under an atmosphere at 37 °C containing 5% CO_2_. The cell cultures were tested for mycoplasms every 3 months and were shown to be always negative.

### Incubation and hypoxia incubation

Cisplatin (Sigma-Aldrich) was dissolved in NaCl 0.9% before each experiment then directly added in the culture medium without serum. All experiment was performed in serum-free CO_2_-independent medium (Thermo Fisher Scientific) supplemented with 500 μM of l-glutamine (Sigma- Aldrich), except for DNA damage repair experiment for which fresh culture medium with serum was added to the cells after cisplatin incubation. For hypoxia experiments, cells were incubated under 1% O_2_, whereas for normoxic conditions cells were incubated under normal atmosphere (20% O_2_).

### Transfection and transduction

For transfection, SQD9 cells were seeded in 75 cm^2^ polystyrene flasks (Corning) at a density of 3,000,000 cells per flask. TMEM45A knockdown was achieved using siGENOMESMART pool human TMEM45A (#M-021085-00 containing a mix of four siRNA: CAAUGUACUUCUGGAGCUA, GGGAAAUGCUGGACAUCUU, AAGCGAACCUGCUAUCUUG, UAAACAAGGUCACUGGAAU from Dharmacon). RISC-free control siRNA purchased from Dharmacon was used as a control for non-specific effects. Cells were transfected 24 h under standard culture conditions with 50 nM siRNA using DharmaFect no. 1 (Dharmacon) transfection reagent according to the manufacturer’s instructions. Cells were then trypsinized and seeded at the appropriate density for further experiments.

For the transduction, cells were seeded in 25 cm^2^ polystyrene flasks (Corning) at a density of 800,000 cells per flask. Cells were then transduced with short-hairpin RNA (shRNA) lentiviral particles (Sigma): empty vector as a control for RCC4 cells and shRNA targeting the luciferase (CCGGCGCTGAGTACTTCGAAATGTCCTCGAGGACATTTCGAAGTACTCAGCGTTTTT) as negative control for SQD9 cells (shCTL) or shRNA targeting TMEM45A mRNA, shRNA22 (CCGGGAGTTCCTTGTTCGGAACAATCTCGAGATTGTTCCGAACAAGGAACTCTTTTTTG) and shRNA 92 (CCGGGATGACTCTAAGTGTACTGTTCTCGAGAACAGTACACTTAGAGTCATCTTTTTTG). The transduction was performed with a multiplicity of infection (MOI) of 5 in fresh medium with serum containing protamine sulfate (0.06 mg/mL) (Sigma-Aldrich) for RCC4 cells. Twenty-four hours after the transduction, the medium was replaced by fresh medium supplemented with puromycin (4 μg/mL) (Sigma-Aldrich) for 6 days of selection. After the selection, cells were seeded in 75 cm^2^ polystyrene flasks (Corning) with medium without puromycin and used for further experiments.

### RNA sequencing

#### Sample preparation

SQD9 cells, untreated or treated with 20 µM of cisplatin for 24 h, were collected and lysed in RLT Lysis Buffer (Qiagen). Total RNA was extracted using the RNeasy Mini Kit (Qiagen) and the automation QIAcube (Qiagen). The analysis was performed for four biological independent experiments. The quality and integrity of the RNA samples was checked using the Bioanalyser 2100 (Agilent). Samples were sent to Genomics core (KU Leuven) for sequencing: a paired-end (PE) library was constructed using TruSeq RNA Library Prep Kit v2 (Illumina). A gene level single-read sequencing was performed on Illumina’s HiSeq 2500 platform (short read 50-SR50).

#### Data analysis

Bioinformatics Consultancy Service of Genomic core performed the data analysis.

- Mapping and differential expression (DE): quality control of raw reads was performed with FastQC v0.11.5 and adapters were filtered with ea-utils v1.2.2.18. The QC made using FastQC permits to determine the GC content of all reads and the sequencing quality base per base. Splice-aware alignment was performed with TopHat v2.0.13 against the human reference genome GRCh37/hg19 (http://hgdownload.soe.ucsc.edu/downloads.html#human) with the following parameters: two allowed mismatches, reads that mapped to more than one site to the reference genome discarded and a minimal score of alignment quality included in count analysis of 10.
- Gene count and differential expression: resulting SAM and BAM alignment files were handled with Samtools v0.1.19.24. Quantification of reads per gene was performed with HT-Seq count v0.5.3p3. Count-based differential expression analysis was done with R-based (The R Foundation for Statistical Computing, Vienna, Austria) Bioconductor package DESeq. DESeq is a method based on negative binomial distribution modeling, that improves the specificity and sensitivities, as well as good control of false-positive errors, taking into account gene length for the counting (https://support.bioconductor.org/p/74572/). Reported *p*-values were adjusted for multiple testing with the Benjamini–Hochberg procedure, which controls false discovery rate (FDR) and differentially expressed genes were selected at a FDR 0.1.
- Gene ontology analysis: gene set enrichment analysis (GSEA-http://software.broadinstitute.org/gsea/msigdb/index.jsp) was performed from count files obtain with HT-Seq count. C5 collection was used to analyze the count datas. The gene sets in the C5 collection are based on GO terms (go-basic.obo, downloaded on 3 May, 2016) and their associations to human genes (gene2go, downloaded from NCBI FTP server on 3 May, 2016). GO are divided in three types: molecular function (MF), cellular component (CC), or biological process (BP). (Jaccard’s coefficient > 0.85-http://geneontology.org/page/guide-go-evidence-codes). Panther (http://www.pantherdb.org/) and Babelomics (http://babelomics.bioinfo.cipf.es/) Gene ontology were used to identify pathways involved from differentially expressed gene lists, selected using an FDR of 0.1. The results obtained were compared to highlight the most relevant pathways/genes of interest.

The RNAseq data were made available: experiment can be viewed in ArrayExpress under this link: https://www.ebi.ac.uk/arrayexpress/experiments/E-MTAB-7518

### Real-time quantitative polymerase chain reaction (RT-qPCR)

For patient biopsies, samples were lyzed in Qiazol reagent. After 5 min at room temperature, 200 μL of chloroform were added and the samples were shacked vigorously for 2–3 min. After centrifugation at 12,000 x *g* for 15 min at 4 °C, the upper aqueous phase was transferred to a new tube and the total RNA was extracted using the RNeasy Mini Kit (Qiagen) and the QIAcube (Qiagen). For the amplification complementary DNA (cDNA) was diluted at 1:100 in MilliQ water and added to the mix reaction containing 300 nM of forward and reverse primers (Table 1) and SYBR Select Master Mix (Thermo Fisher Scientific) in a 5 to 1 ratio. qPCR was conducted on a StepOnePlus system (Applied Biosystems) following thermal cycling: 95 °C for 5 min followed by 40 cycles at 95 °C for 30 s and 60 °C for 1 min. mRNA expression level was quantified using the threshold cycle method, given the fold change (FC): downregulated genes with a FC < 0.5 and upregulated genes with a FC > 1.5.

**Table 1:** Primers used for qPCR and PCR.

Genes	Firm	Forward primer	Reverse primer
*23* *kDa*	Integrated DNA Technologies	GCCTACAAGAAAGTTTGCCTATCTG	TGAGCTGTTTCTTCTTCCGGTAGT
*ATF6B*	Integrated DNA Technologies	AGAGTCCCTGTCCCCTTCAG	ACTACAGGTTTGGGCTGCAG
*CAIX*	Integrated DNA Technologies	GAGGCCTGGCCGTGTTG	AACTGCTCATAGGCACTGTTTTCTT
*Calreticulin*	Integrated DNA Technologies	CACCAACGATGAGGCATACG	TTTGTTTCTCTGCTGCCTTTGTTAC
*EYA3*	Integrated DNA Technologies	AGCCATGGTTCATCTGTGGG	CAGTGCTTCCTTCCTCTGGG
*HSP90B1*	Integrated DNA Technologies	TCATCCAACTGACATTACTAGCC	ACAAATGGAGAAGATTCAGCCTC
*MANF*	Integrated DNA Technologies	ACCAGGACCTCAAAGACAGAG	CATTGATGATTTTGGTGGCTGC
*PDIA6*	Integrated DNA Technologies	GAAGATGTTTGGATGGTTGAGTTCT	TAGCATCCACAGCTGCCAGTT
*TMEM45A*	Integrated DNA Technologies	TTATGCAGTAACCATTGTCATCGTT	TGATTCTTGTTCTCGTTCAGCATT
*XBP1*	Integrated DNA Technologies	GATGGATGCCCTGGTTGCT	TGAAGAGTCAATACCGCCAGA

For cells samples, after the incubation, 600 μL of RLT Lysis Buffer (Qiagen) was added to lyse the cells. Total RNA was extracted using the RNeasy Mini Kit (Qiagen) and the QIAcube (Qiagen). mRNA contained in 2 μg of total RNA was reverse transcribed using first Strand cDNA Synthesis kit (Roche Life Science) following supplier’s instructions. For the amplification cDNA was diluted at 1:100 in MilliQ water and added to the mix reaction containing 300 nM of forward and reverse primers (Table 1) and SYBR Select Master Mix (Thermo Fisher Scientific) in a 5 to 1 ratio. qPCR was conducted on a StepOnePlus system (Applied Biosystems) following thermal cycling: 95 °C for 5 min followed by 40 cycles at 95 °C for 30 s and 60 °C for 1 min. mRNA expression level was quantified using the threshold cycle method. The experiment was performed for 3 biological independent experiments.

### Western blot

After the incubation, cells were lysed in lysis buffer solution containing Trizma 40 mM (TRIS base) (Sigma-Aldrich), KCL 150 mM (Merck Millipore) and Tritriplex III 1 mM (Merck Millipore) with MilliQ water, Triton X100 1% (Sigma-Aldrich), phosphatase inhibitor cocktail (PIC, Roche), and phosphatase inhibitor buffer (PIB containing sodium orthovanadate (Na_3_VO_3_) (Sigma-Aldrich), 4-nitrophenylphosphate (PNPP) (Sigma-Aldrich), β-glycerophosphate (VWR) and sodium fluoride (NaF) (Merck)). Then 15 μg of proteins were mixed with MilliQ water and blue sample buffer composed of DTT (dithiothreitol) 5% and LDS (lithium dodecyl sulfate) sample buffer 4 × (Thermo Fisher Buffer) and heated up during 10 min to 70 °C. Proteins were then separated on NuPAGE 4–12% Bis-Tris Gel or 3–8% Tris Acetate Gel (Thermo Fisher Scientific). Afterwards, proteins were transferred on a PVDF (polyvinylidene fluoride) membrane (Thermo Fisher Scientific). The membrane was incubated overnight at 4 °C with the primary antibody (Table 2) in Odyssey Blocking buffer (Li-Cor Biosciences) with 0.1% of Tween 20 (Bio-Rad Laboratories) and incubated with the secondary antibody during 1 h in Odyssey Blocking buffer (Li-Cor) with 0.1% of Tween 20 (Bio-Rad Laboratories). The membrane was scanned using the Odyssey Infrared Imager (Li-Cor Biosciences). The fluorescence was quantified using the imaging software Odyssey V3.0 from the Odyssey Infrared Imager (Li-Cor Biosciences).

**Table 2:** Antibodies used for immunofluorescence labeling and western blot.

Protein	Primary antibody	Secondary antibody
Immunofluorescence labeling		
TMEM45A	Anti-TMEM45A (Rabbit, Sigma HPA024082, 1/250)	Alexa 488 nm (Anti-rabbit, Thermo Fisher Scientific A-11008, 1/1000)
Golgin-97	Anti-Golgin-97 (Mouse, Thermo Fisher Scientific A-21270, 1/400)	Alexa 568 nm (Anti-mouse, Thermo Fisher Scientific, A-11004, 1/1000)
GM130	Anti-GM130 (Mouse, BD Biosciences 610823, 1/250)	Alexa 568 nm (Anti-mouse, Thermo Fisher Scientific, A-11004, 1/1000)
RAD51	Anti-RAD51 (Rabbit, Sigma HPA024012, 1/400	Alexa 488 nm (Anti-rabbit, Thermo Fisher Scientific A-11008, 1/1000)
H_2_AX	Anti-Phospho Histone H_2_A.X S139 (Rabbit, Cell Signaling #2577S, 1/200)	Alexa 488 nm (Anti-rabbit, Thermo Fisher Scientific A-11008, 1/1000)
Western blot		
PARP	Anti-PARP (Mouse, BD Biosciences 6639GR, 1/2000)	IRDye 680RD (Anti-mouse, Li-Cor Biosciences 926–68070, 1/10,000)
Caspase 3	Anti-Caspase 3 (Rabbit, Cell Signaling #9662S, 1/1000)	IRDye 800CW (Anti-rabbit, Li-Cor Biosciences 926–32211, 1/10,000)
H_2_AX	Anti-Phospho Histone H_2_A.X S139 (Rabbit, Cell Signaling #2577S, 1/1000)	IRDye 800CW (Anti-rabbit, Li-Cor Biosciences 926–32211, 1/10,000)
ATM/ATR substrate	Anti-Phospho ATM/ATR substrate S*Q (Rabbit, Cell Signaling #9607, 1/1000)	IRDye 800CW (Anti-rabbit, Li-Cor Biosciences 926–32211, 1/10,000)
EYA3	Anti-EYA3 (Rabbit, Abcam, ab129501, 1/1000)	IRDye 800CW (Anti-rabbit, Li-Cor Biosciences 926–32211, 1/10,000)
IRE1α total	Anti-IRE1α (Rabbit, Novus bilogicals, NB100-2324, 1/1000)	IRDye 800CW (Anti-rabbit, Li-Cor Biosciences 926–32211, 1/10,000)
Phospho IRE1α	Anti-IRE1α Phospho S724 (Rabbit, Abcam, ab124945, 1/1000)	IRDye 800CW (Anti-rabbit, Li-Cor Biosciences 926–32211, 1/10,000)
β-actin	Anti-β-Actin (Mouse, Sigma-Aldrich A5441, 1/10,000)	IRDye 680RD (Anti-mouse, Li-Cor Biosciences 926–68070, 1/10,000)

### MTT

After incubation, MTT (Thiazol Blue Tetrazolium Bromide) (Sigma-Aldrich) was diluted in PBS at 2.5 mg/mL and added to cell media for 2 h at 37 °C. Afterwards, cells were lysed with a lysis buffer composed of sodium dodecyl sulfate (SDS) 30% (Merck Millipore) and N, N-dimethylformamide (Sigma- Aldrich) in a 2 to 1 ratio at pH 4.7 during an hour at 37 °C. Finally, the optic density was read with a spectrophotometer at 570 nm.

### Caspase 3/7 activity

After incubation, the cells were lysed with a lysis buffer containing HEPES 20 mM (Sigma-Aldrich), Tritriplex III 4 mM (Merck Millipore), CHAPS 0.2% (3-[(3-cholamidopropyl)dimethylammonio]-1-propanesulfonate hydrate) (Sigma-Aldrich), sucrose 300 mM, DTT 1 M (Sigma-Aldrich), and MilliQ water. Then, reaction buffer composed of PIPES (1,4-Piperazinediethanesulfonic acid**)** 40 mM (Sigma-Aldrich), NaCl 200 mM (Merck Millipore), Tritriplex III 2 mM (Merck Millipore), CHAPS 0.2% (Sigma-Aldrich), sucrose 300 mM, DTT 1 M (Sigma-Aldrich) was added to the lysis solution containing 15 μg of proteins. The fluorogenic substrate Ac-DEVD-AFC (BD Pharmingen) was used to measure caspase 3 and 7 activity. Fluorescence intensity was measured using a spectrophotometer at 400 nm for excitation and 505 nm for emission.

### LDH release

After the incubation, the supernatant of each well was collected in 1.5 mL microtubes labeled as “Pellet”. Two-hundred fifty microliters of PBS-Triton 4% were added in each well and plates were left at room temperature with a slight agitation during 10 min. The “Pellet” microtubes were centrifuged during 5 min at 2000 rpm at 4 °C. The supernatant of the “Pellet” microtubes was then transferred into microtubes called “Supernatant” while the pellets were resuspended in 250 μL of PBS-Triton 4%. The cell lysate in the wells of the plates was collected in a third series of microtubes labeled as “Lysate”.

One-hundred microliters of each sample were placed in a 96-well plate, except for the “Lysate” samples of which 5 μL were diluted in 95 μL of PBS-Triton 4%. One-hundred microliters of Cytotoxicity Detection Kit (Roche) were added in each well and after 15 min of reaction, the optic density was read with a spectrophotometer at 490 nm and 655 nm every 15 min until at least one value reached 2. The signal obtained at 655 nm was subtracted of the signal obtained at 490 nm and the percentage of cytotoxicity was then calculated as follows:

$${\it{\% }}\;{\mathrm{cytotoxicity}} = \frac{{\left( {{\mathrm{Pellet}} - {\mathrm{Blank}}} \right) + \left( {\left( {{\mathrm{Supernatant}} - {\mathrm{Blank}}} \right) \ast 4} \right)}}{{\left( {{\mathrm{Pellet}} - {\mathrm{Blank}}} \right) + \left( {\left( {{\mathrm{Supernatant}} - {\mathrm{Blank}}} \right) \ast 4} \right) + \left( {\left( {{\mathrm{Lysate}} - {\mathrm{Blank}}} \right) \ast 20} \right)}}$$

### Immunofluorescence labeling

Cells were seeded on glass coverslips in 24-well plates (Costar) at a density of 25,000 cells per well. After the incubation time, the medium was removed and cells were then fixed during 10 min with 4% paraformaldehyde (PFA) in phosphate buffer saline (PBS) before being washed three times with PBS and permeabilized with PBS-triton 1% for 5 min at room temperature. After permeabilization, cells were washed three times for 10 min with PBS-BSA 2% (Bovine Serum Albumin) (Santa Cruz Biotechnology) then incubated with the primary antibody overnight at 4 °C in the dark (Table 2). The next day, cells were washed three times with PBS-BSA 2% before being incubated for 1 h with the secondary antibody and Hoechst (Thermo Fisher Scientific H-21491) at 2 μg/mL at room temperature in the dark. Thereafter, cells were washed three times with PBS-BSA 2% and once with PBS. The coverslips were finally mounted on slides in Mowiol mounting solution (Sigma) warmed at 57 °C. Slides were observed by confocal microscopy (SP5, Leica).

### Statistical analysis

**Fig. 1:**
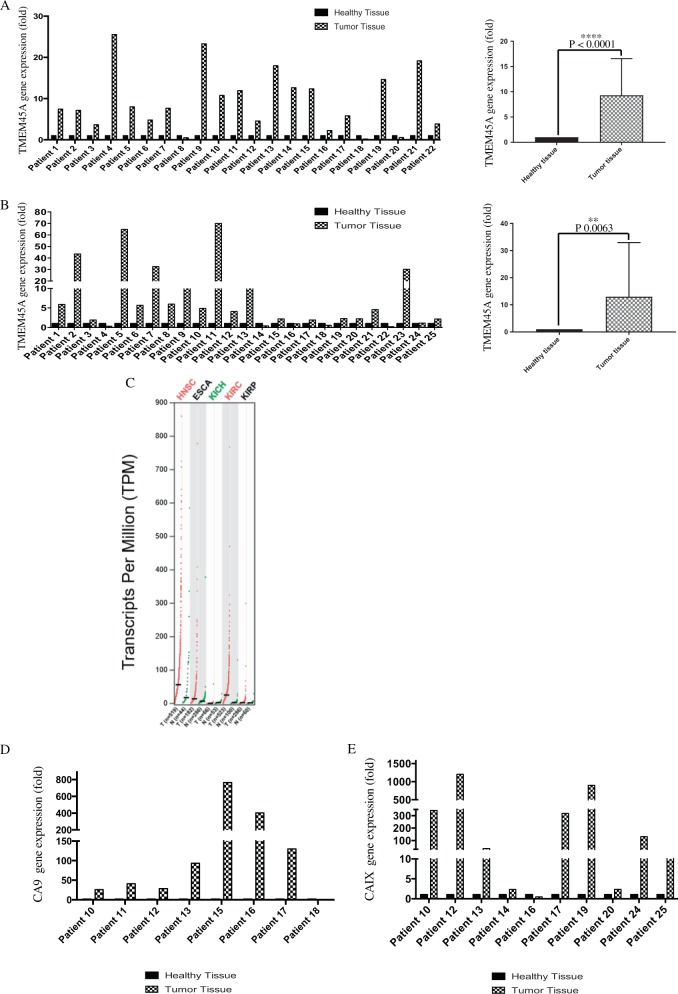
TMEM45A and CAIX expression in head and neck and renal cancers. **a** The expression level of TMEM45A was determined by RT-qPCR in 18 pairs of head and neck cancer biopsies and corresponding adjacent normal tissues. In the right panel, results are expressed as mean ± SD (*n* = 22). **b** The expression level of TMEM45A was determined by RT-qPCR in 25 pairs of renal cancer and corresponding adjacent normal tissues. In the right panel, results are expressed as mean ± SD (*n* = 25). **c** TCGA analysis of samples from human tumors (red) and corresponding healthy tissues (green). ESCA esophageal carcinoma, HNSC head and neck squamous carcinoma, KICH kidney chromophobe, KIRC kidney renal clear cell carcinoma, KIRP kidney renal papillary cell carcinoma. The number of samples is given between brackets, red labeling indicates a significant increase in TMEM45A expression in two cancer types. The expression level of CAIX was determined by RTqPCR (**d**) in eight pairs of head and neck cancer biopsies and corresponding adjacent normal tissues and (**e**) in ten pairs of renal cancer biopsies and corresponding adjacent normal tissues. **p < 0.01, ***p < 0.001.

Prism software was used for the generation of all statistical analyses. Paired *T*-test with two tailed *p*-value was used for the Fig. 1. Two-way ANOVA and multiple comparisons were used for all other figures. *p* < 0.05 was considered statistically significant. The variance was tested with Prism for all experiments and shown to be similar between the groups within one experiments. For consistency in these comparisons, the following identifies the significance values: *p *<* 0.05, **p < 0.01, ***p < 0.001, ****p < 0.0001. All results are presented from at least three independent replicates.

## Results

### TMEM45A expression in HNSCC and ccRCC human biopsies

To explore TMEM45A expression in human samples of HNSCC or ccRCC patients, *TMEM45A* mRNA level was evaluated by RT-qPCR in tumor samples and corresponding adjacent healthy tissues for each patient. *TMEM45A* transcript was upregulated in tumor tissues compared to healthy tissues in 86% (19/22) and 76% (19/25) of HNSCC and ccRCC samples respectively (Fig. 1a, b). Furthermore, TCGA analysis showed that TMEM45A expression was significantly higher in HNSCC and ccRCC human tumors than in corresponding healthy tissues (Fig. 1c). *TMEM45A* is upregulated in hypoxic conditions under the control of the transcription factor HIF1 (hypoxia inducible factor 1)^12^. Furthermore, in normoxic conditions, HIF1α stability is regulated by pVHL. Since pVHL is mutated in ccRCC, HIF1α is no longer degraded, hence conferring a state of pseudo-hypoxia^20^. It has to be noted that, in most studies, HIF1 was shown to suppress while HIF2 was shown to promote tumor growth. In order to sought whether HIF1 was activated in these samples, we analyzed the expression of a second HIF1 target gene, *CAIX* (Carbonic Anhydrase IX). All HNSCC samples, which displayed *TMEM45A* overexpression also presented *CAIX* upregulation. For ccRCC, 9 samples out of 10 showed the same expression profiles for *TMEM45A* and *CAIX* (Fig. 1d, e). These data revealed that *TMEM45A* is upregulated in a majority of patients with HNSCC and ccRCC and that this upregulation is probably under the control of HIF1.

### Identification of deregulated genes and associated signaling pathways in TMEM45A-inactivated cells

**Fig. 2:**
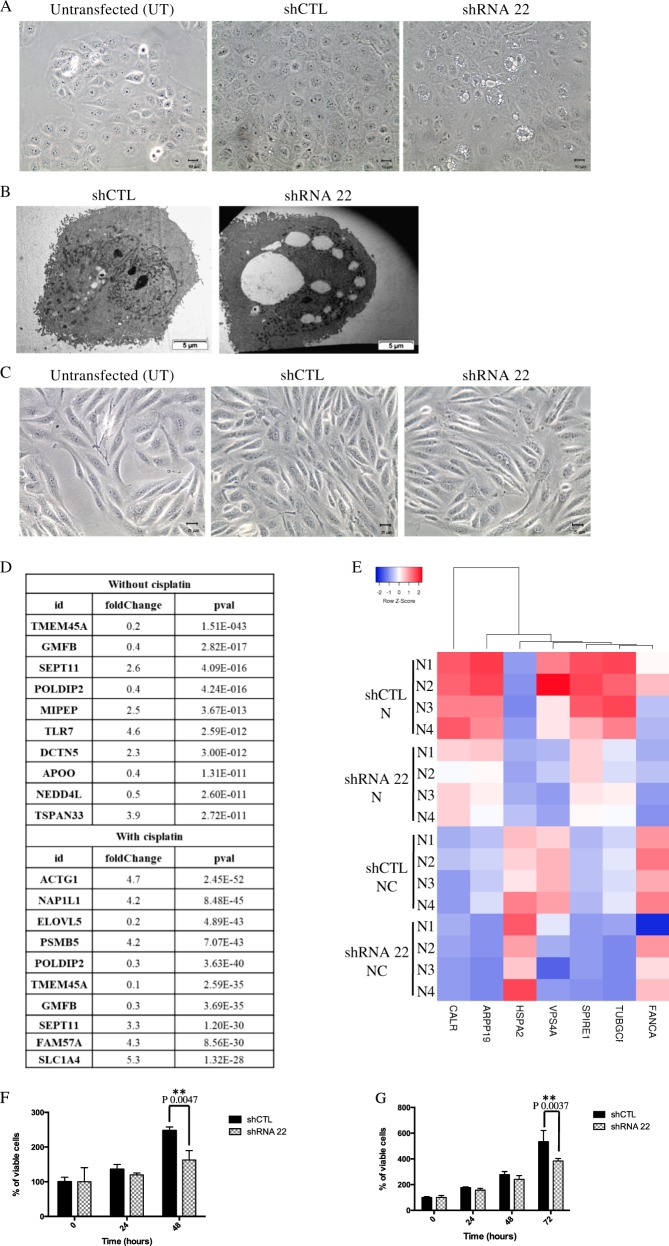
Effect of TMEM45A inactivation on cell morphology and proliferation in SQD9 and RCC4 cells. SQD9 and RCC4 cells were transduced with lentiviral particles expressing shRNA control (shCTL) or shRNA targeting the mRNA of TMEM45A (shRNA22) or were not transduced (UT). **a** SQD9 cell morphology was studied by optical microscopy (magnification ×200) and **b** transmission electron microscopy. **c** RCC4 cell morphology was studied by optical microscopy (magnification ×200). **d**, **e** SQD9 cells were transduced with lentiviral particles expressing shRNA control (shCTL) or shRNA targeting the mRNA of TMEM45A (shRNA 22). The cells were incubated with or without 100 mM of cisplatin in normoxic (N) conditions for 24 h and gene expression level was assessed by RNA sequencing. **d** Ten first genes differentially expressed in the knockdown cells in the absence or in the presence of cisplatin. **e** The transcriptome analysis was performed using Babelomics and GSEA after RNA sequencing. Heatmap of differentially expressed genes produced by using the heatmapper^26^ (http://www.heatmapper.ca) showed a potential deregulation of the G2/M transition pathway. Genes have been reordered by the method of average clustering. A dendrogram is shown for the clustering of genes. Genes with relatively high expression are marked in red, genes with relatively low expression are marked in blue. The corresponding values are presented in Supplementary Fig. 4. **f** After the tranduction, SQD9 cells were incubated in normoxic conditions for 24 and 48 h then, the proliferation was assessed by MTT assay. The signal intensity of the control cells at the seeding (time 0) was used to normalize the number of viable cells at different time points. Results are expressed as mean ± SD (*n* = 3). **p < 0.01. **g** After the transduction, RCC4 cells were incubated in normoxic conditions for 24, 48, and 72 h then, the proliferation was assessed by MTT assay. The signal intensity of the control cells at the seeding (time 0) was used to normalize the number of viable cells at different time points. Results are expressed as mean ± SD (*n* = 3). N normoxia, NC normoxia + cisplatin. **p < 0.01.

In order to investigate the role of TMEM45A in cancer cell chemoresistance, cisplatin sensitivity was determined for different cancer cell lines: SQD9 cells for HNSCC and RCC4 + pVHL for ccRCC. Cisplatin is a chemotherapeutic drug inducing DNA damage during cell replication, leading to cell death by apoptosis^21^. The IC50 was reached after incubation with 100 μM for 24 h for SQD9 cells and with 20 μM for 48 h for RCC4 + pVHL cells (Supplementary Fig. 1). These conditions were thus used for the next experiments. Then, to explore the functions of TMEM45A, its expression was inactivated using siRNA or shRNA strategies. A strong decrease in both TMEM45A mRNA and protein levels was observed in both cell lines for both inactivation strategies (Supplementary Fig. 2). TMEM45A inactivation was associated with the presence of large vesicles in SQD9 cells (Fig. 2a, b), whereas no change in morphology was observed for RCC4 + pVHL cells (Fig. 2c).

In order to identify putative pathways deregulated upon TMEM45A inactivation, a transcriptome analysis by RNA sequencing was performed in SQD9 cells. Two-hundred sixty-three differentially expressed genes were identified in the absence of cisplatin and 469 differentially expressed genes were identified in the presence of cisplatin (Fig. 2d). Unsurprisingly, the most downregulated gene in cells incubated without cisplatin was *TMEM45A*, with a fold change of 0.2. Another interesting gene was *POLDIP2*. POLDIP2 has been shown to regulate in DNA polymerase activation^22^ and to be an oxygen-sensitive protein^23,24^. Furthermore, POLDIP2 has been implicated in tumor growth and invasiveness in non-small cell lung cancer^25^. All these data made of POLDIP2 a good candidate for further investigation but the difference observed in mRNA expression was not confirmed at protein level (data not shown).

In order to identify pathways affected by TMEM45A knockdown, bioinformatic analysis using Babelomics analysis and Gene Set Enrichment Analysis (GSEA) was performed. Then, heatmaps of differentially expressed genes for these pathways were generated using Heatmapper^26^. Several pathways appeared to be deregulated such as the G2/M transition pathway (Supplementary Fig. 3 and Fig. 2e). In parallel, a decrease of about 30% in the proliferation rate for both SQD9 and RCC4 + pVHL cells was observed in the knockdown cells compared to control cells (Fig. 2f, g). This decrease in proliferation is probably due to a delay in cell cycle progression since an accumulation of cells in S phase was detected (Supplementary Fig. 5). These data indicate a role of TMEM45A in the regulation of cell proliferation.

### TMEM45A inactivation impacts the sensitivity to cisplatin

**Fig. 3:**
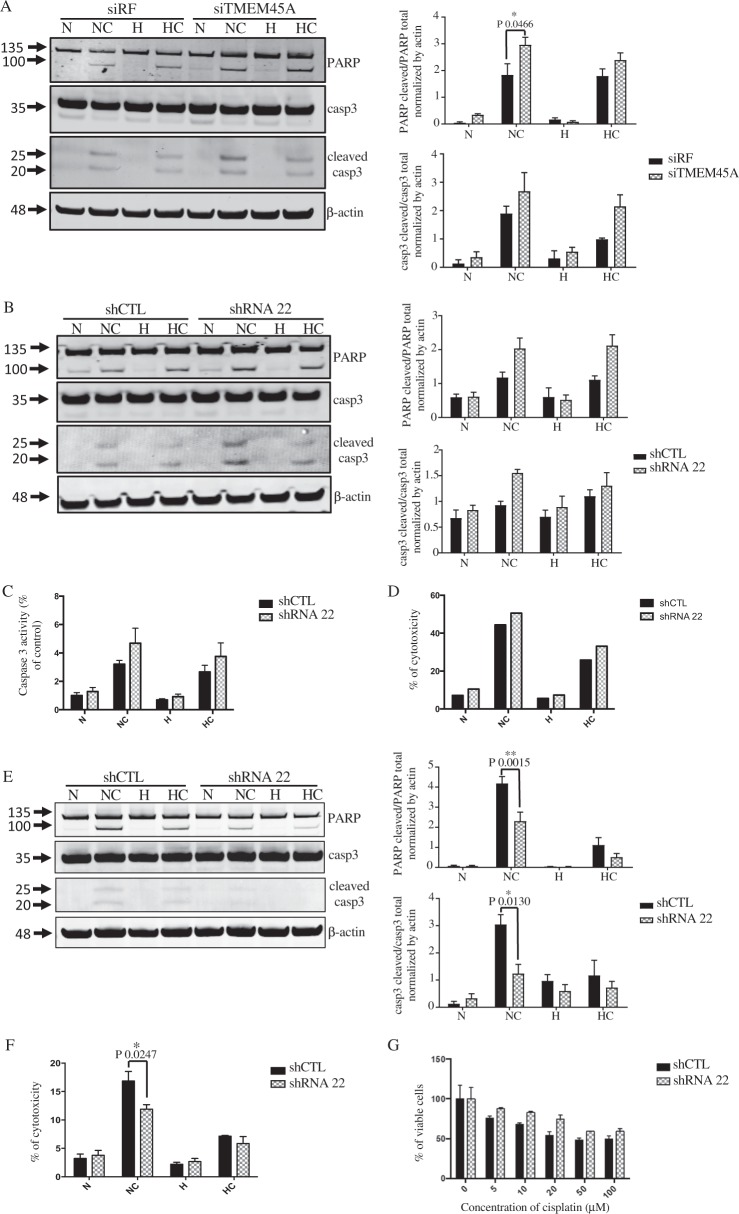
Effect of TMEM45A inactivation on the chemosensitivity to cisplatin in SQD9 and RCC4 cells. SQD9 cells were transfected with siRNA control (siRF = risc free) or siRNA targeting the mRNA of TMEM45A (siTMEM45A). SQD9 cells were incubated with or without 100 mM of cisplatin in normoxic (N) and hypoxic (H) conditions for 24 h. **a** The apoptosis was studied by western blot analyses of PARP and caspase 3 cleavage. Actin was used as the loading control. Results are expressed as mean ± SD (*n* = 3). **b**–**d** SQD9 cells were transduced with lentiviral particles expressing shRNA control (shCTL) or shRNA targeting the mRNA of TMEM45A (shRNA 22). SQD9 cells were incubated with or without 100 mM of cisplatin in normoxic (N) and hypoxic (H) conditions for 24 h. **b** After transduction and incubation, the cleavage of PARP and caspase 3 was assessed by western blot. Actin was used as the loading control. Results are expressed as mean ± SD (*n* = 3). **c** After the incubation, caspase 3 and 7 activity was assessed by measuring free AFC released from the cleavage of caspase 3 and 7 specific substrate Ac-DEVD-AFC. Results are expressed as mean ± SD (*n* = 5). **d** The cytotoxicity of cisplatin was assessed by LDH release. Results are representative of three biological replicates. **e**–**g** RCC4 cells were transduced with lentiviral particles expressing shRNA control (shCTL) or shRNA targeting the mRNA of TMEM45A (shRNA 22). RCC4 cells were incubated with or without 20 mM of cisplatin in normoxic (N) or hypoxic (H) conditions for 48 h. **e** After transduction and incubation, the cleavage of PARP and caspase 3 was assessed by western blot. Actin was used as the loading control. Results are expressed as mean ± SD (*n* = 3). **f** The cytotoxicity of cisplatin was assessed by LDH release. Results are expressed as mean ± SD (*n* = 3). **g** After the incubation, the number of viable cells was determined using MTT assay. The signal intensity of the control cells was used for normalization a. Results re expressed as mean ± SD (*n* = 3). N normoxia, NC normoxia + cisplatin, H hypoxia, HC hypoxia + cisplatin. *p < 0.05, **p < 0.01.

Several deregulated pathways in TMEM45A knockdown cells were related to apoptosis (Supplementary Fig. 3). To study the potential implication of TMEM45A in chemosensitivity, cisplatin-induced apoptosis was investigated in TMEM45A knockdown cells. In SQD9 cells, after TMEM45A silencing using siRNA, the cells were incubated with or without 100 μM of cisplatin in normoxic or hypoxic conditions and the cleavage of two effectors of apoptosis, PARP (Poly(ADP-ribose) polymerase) and caspase 3, was analyzed by western blot. Figure 3a shows that the cleavage of the two effectors was much higher in the inactivated cells compared to control cells when incubated in the presence of cisplatin both in normoxic and hypoxic conditions. In order to validate the impact of TMEM45A inactivation on chemosensitivity, TMEM45A expression was also silenced using shRNA. As for siRNA, the cleavage of the two effectors was more important in the TMEM45A-inactivated cells compared to control cells (Fig. 3b) meaning that, when TMEM45A was absent, SQD9 cells were more sensitive to cisplatin. An increase in caspase 3/7 activity in the TMEM45A-inactivated cells was also detected, which, however, did not reach statistical significance (Fig. 3c). Furthermore, the cytotoxicity of cisplatin was increased in cells inactivated for TMEM45A (Fig. 3d). In order to confirm this result, TMEM45A overexpression was performed. TMEM45A overexpressing cells were more resistant to cisplatin than control cells (Supplementary Fig. 6A). This was also observed in another HNSCC cell line, Cal 27 (Supplementary Fig. 6B).

All together, these results showed that SQD9 cells were more sensitive to cisplatin in the absence of TMEM45A and indicated that this protein exerts a role in the resistance of SDQ9 cells to this drug.

To confirm the role of TMEM45A in drug resistance, we performed similar experiments in RCC4 + pVHL cells. TMEM45A was knockdown using shRNA and the cells were incubated with or without 20 μM of cisplatin for 48 h. Surprisingly, TMEM45A knockdown led to a significant decrease in the cleavage of PARP and caspase 3 compared to control cells (Fig. 3e). To confirm this result, cisplatin cytotoxicity was assessed. An increase in cytotoxicity and a decrease in cell viability were observed in knockdown cells (Fig. 3f, g). RCC4 + pVHL cells were thus more resistant to cisplatin when TMEM45A was knockdown, meaning that, in this cell type, TMEM45A exerts a pro-apoptotic role.

### Effect of TMEM45A inactivation on cisplatin-induced DNA damage

**Fig. 4:**
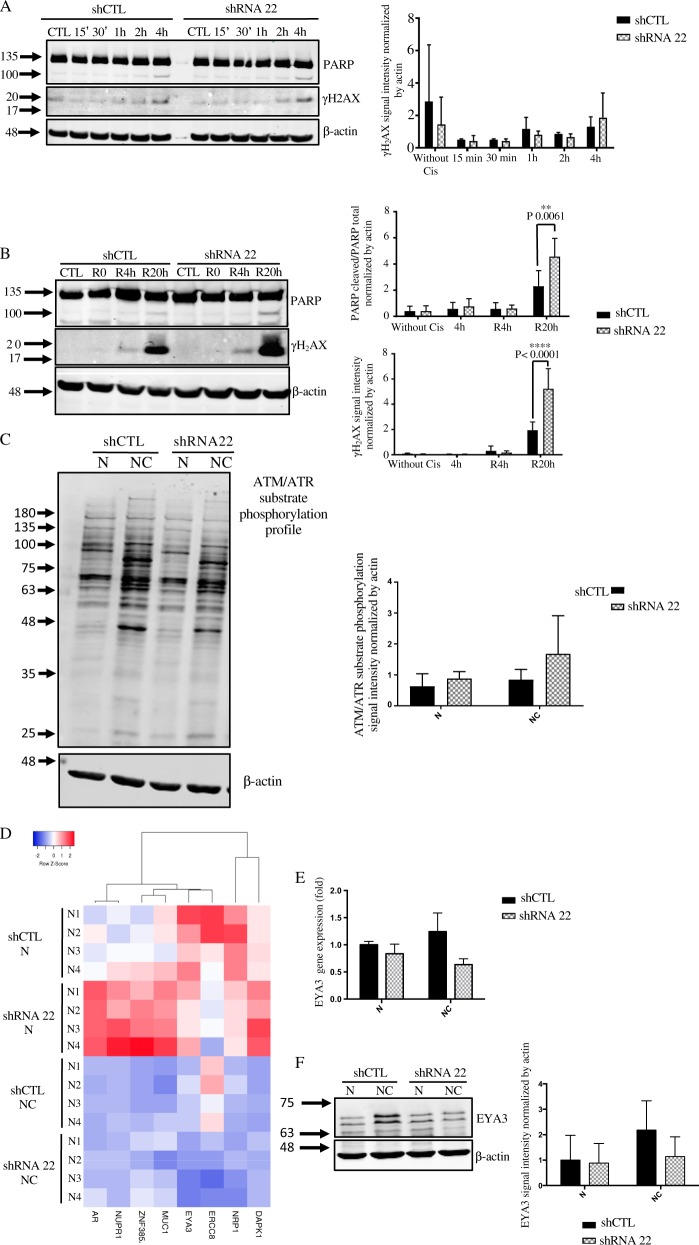
Effect of TMEM45A inactivation on DNA damage induction and repair. SQD9 cells were transduced with lentiviral particles expressing shRNA control (shCTL) or shRNA targeting the mRNA of TMEM45A (shRNA 22). **a** SQD9 cells were incubated with or without 100 mM of cisplatin in normoxic conditions for 15, 30 min, 1, 2, and 4 h. After the incubation, proteins were extracted and PARP cleavage and gH2AX protein level were assessed by western blot. Actin was used as the loading control. Results are expressed as mean ± SD (*n* = 3). **b** SQD9 cells were incubated with or without 100 mM of cisplatin in normoxic conditions for 4 h then the fresh medium without cisplatin was added to the cells for 4 or 20 h. After the incubation, proteins were extracted and PARP cleavage and gH2AX protein level were assessed by western blot. Actin was used as the loading control. Results are expressed as mean ± SD (*n* = 3). **c** SQD9 cells were incubated with or without 100 mM of cisplatin in normoxic conditions for 24 h. ATM/ATR substrate phosphorylation profile was assessed by western blot. Actin was used as loading control. Results are expressed as mean ± SD (*n* = 3). **d** SQD9 cells were transduced with lentiviral particles expressing shRNA control (shCTL) or shRNA targeting the mRNA of TMEM45A (shRNA 22). The cells were incubated with or without 100 mM of cisplatin in normoxic (N) conditions for 24 h and gene expression level was assessed by RNA sequencing. The transcriptome analysis was performed using Babelomics and GSEA after RNA sequencing. Heatmap of differentially expressed genes produced by using Heatmapper^26^ (http://www.heatmapper.ca) showed a potential deregulation of DNA damage and apoptosis activation. Genes have been reordered by the method of average clustering. A dendrogram is shown for the clustering of genes. Genes with relatively high expression are marked in red, genes with relatively low expression are marked in blue. The corresponding values are presented in Supplementary Fig. 4. **e** SQD9 cells were incubated with or without 100 mM of cisplatin in normoxic conditions for 24 h. The expression level of EYA3 was determined by RT-qPCR. Results are expressed as mean ± SD (*n* = 3). **f** SQD9 cells were incubated with or without 100 mM of cisplatin in normoxic conditions for 24 h. EYA3 expression level was assessed by western blot. Actin was used as loading control. Results are expressed as mean ± SD (*n* = 3). N normoxia, NC normoxia + cisplatin. **p < 0.01, ****p < 0.0001.

To identify by which mechanism TMEM45A plays a pro or an anti-apoptotic role according to the cancer cell type, we investigated the impact of cisplatin on DNA damage response. The efficiency of this drug depends not only on its ability to induce DNA damage but also on the cell ability to detect and respond to these damages^27^. Indeed, following DNA damage, cells may either repair the damage or if they cannot repair, the damages activate cell death by apoptosis. Histone H_2_AX (H_2_A Histone Family Member X) is one of the markers for DNA double strand breaks and allows, by its phophorylation (then named γH_2_AX), the detection of DNA damage and the recruitment of DNA damage repair proteins^28^. Thus, we investigated the detection and repair of DNA damage in the two cell lines. SQD9 cells were incubated in the presence of cisplatin for different durations and the abundance of PARP fragment and of γH_2_AX was studied for apoptosis and DNA damage detection respectively. Figure 4a shows an increased level of γH_2_AX after 1, 2, and 4 h of treatment but no difference was observed in knockdown cells compared to control cells. These results suggest that TMEM45A inactivation had no effect on the DNA damage induction or detection. Furthermore, for these incubation times, the cleavage of PARP observed was very low, suggesting that apoptosis was not yet activated.

Next, we explored DNA damage during recovery to analyze repair ability. Cells were incubated in the presence of cisplatin. The drug was thenremoved and cells were incubated in fresh medium. After 4 h and 20 h of recovery, γH_2_AX reappeared and was significantly increased in the knockdown cells compared to control cells. The same results were observed for PARP cleavage, suggesting that SQD9 cells underwent apoptosis after 4 h and 20 h of recovery (Fig. 4b). TMEM45A inactivation in SQD9 cells using another shRNA led to similar results (Supplementary Fig. 7).

It has to be noted that γH_2_AX is also present at DNA points breaks when DNA is fragmented during apoptosis^29^. So, a part of γH_2_AX observed was probably due to apoptosis. There are several possibilities to explain these results. First, even if cisplatin was removed from the medium, the drug could be sequestrated inside the cells and could continue to produce DNA damage. No difference in DNA damage induction or detection was observed. Furthermore, we analyzed the phosphorylation of ATM substrate. ATM is recruited and activated by DNA double strand breaks and phosphorylates several targets such as H_2_AX^30,31^. Figure 4c shows no difference in ATM/ATR substrate phosphorylation profile in the knockdown cells compared to control cells. These results confirm that TMEM45A inactivation did not impact the induction or the recognition of DNA damage. The second possibility is a decrease in the ability of the cells to evade cisplatin-induced cell death for example via the regulation of proteins involved in the apoptotic pathway. Indeed, deregulation of the expression of repair protein encoding genes was observed in the RNA-sequencing analysis (Supplementary Fig. 3 and Fig. 4d). The third possibility is a defect in DNA damage repair mechanism in the knockdown cells, hence leading to cell death. In order to confirm this hypothesis, we explored the possible role of TMEM45A in the recruitment of DNA damage repair proteins. RNA-sequencing data showed that EYA3 (Eyes Absent Homolog 3) was differentially expressed in the knockdown cells in the presence of cisplatin. EYA3 is known to interact with γH_2_AX only under DNA damage conditions. The phosphatase activity of EYA3 dephosphorylates γH_2_AX, leading to DNA damage repair and cell survival^32^. EYA3 downregulation both at mRNA and protein levels was observed when TMEM45A was silenced (Fig. 4e, f). In addition, *EYA3* expression was assessed in head and neck cancer biopsies. The results show that the expression profiles of *TMEM45A* and of *EYA3* were similar. Indeed, when *TMEM45A* was upregulated *EYA3* was also overexpressed and the only patient who presented a downregulation of *TMEM45A* also displayed a downregulation of *EYA3* (Supplementary Fig. 8). These results suggest that TMEM45A silencing had an impact on DNA damage repair through a lack of dephosphorylation of γH_2_AX by EYA3, hence inducing a decrease in the recruitment of proteins involved in DNA damage repair and, consequently, cell death.

**Fig. 5:**
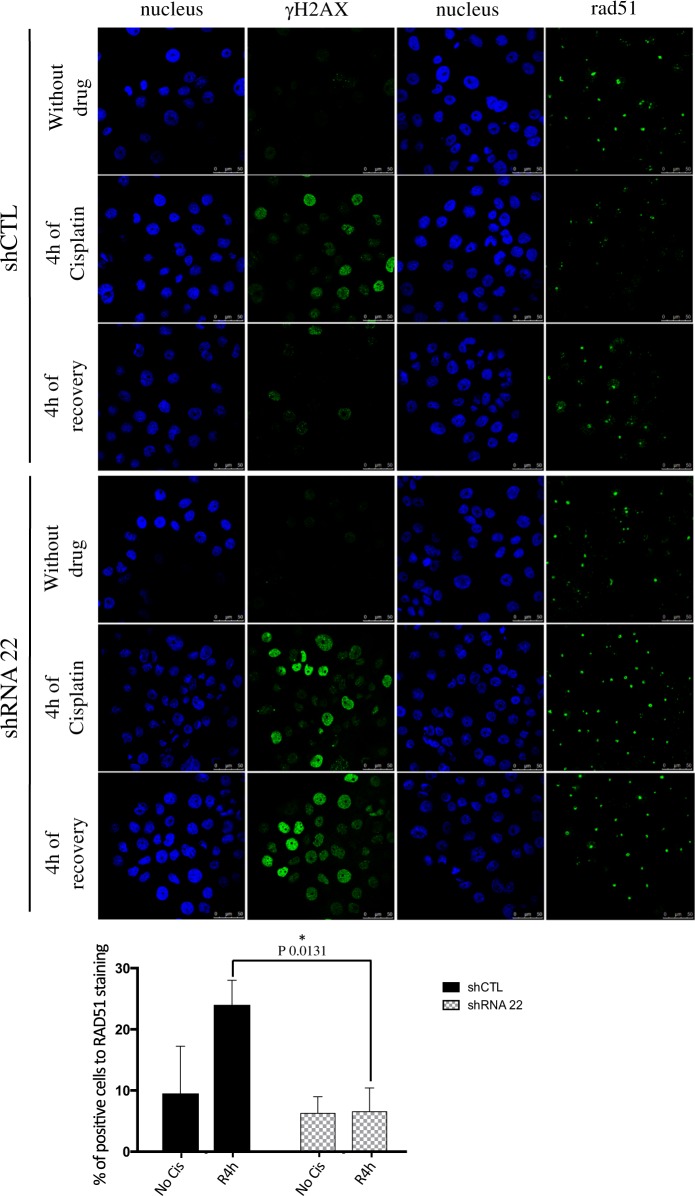
Effect of TMEM45A inactivation on the recruitment of RAD51 to DNA damage foci. SQD9 cells were transduced with lentiviral particles expressing shRNA control (shCTL) or shRNA targeting the mRNA of TMEM45A (shRNA 22). SQD9 cells were incubated with or without 100 mM of cisplatin in normoxic conditions for 4 h then fresh medium without cisplatin was added to the cells for 4 h. After the incubation, the recruitment of RAD51 was studied by confocal microscopy using anti-RAD51 antibody and Alexa 488 anti-rabbit antibody and the abundance of gH2AX was studied using anti-gH2AX antibody and Alexa 488 anti-rabbit antibody. The nucleus was detected with Hoechst. Scale bars: 50 μm. The RAD51 signal intensity was quantified by Image J on more than 100 cells. Results are expressed as mean ± SD (*n* = 3). *p < 0.05.

To confirm the role of TMEM45A in DNA damage repair, the recruitment of one of the major proteins involved in DNA damage repair, RAD51 (RAD51 recombinase), was studied. γH_2_AX and RAD51 immunofluorescence labeling showed a strong decrease in the recruitment of RAD51 to DNA damage foci in TMEM45A knockdown cells exposed to cisplatin. Furthermore, the absence of RAD51 recruitment was associated with the presence of γH_2_AX labeling meaning that the DNA damage was still present and detected (Fig. 5). These results indicate that the absence of TMEM45A increased SQD9 cell chemosensitivity by decreasing RAD51-associated DNA damage repair. All these experiments were repeated in RCC4 + pVHL cells but no differences in DNA damage induction or repair were observed (data not shown).

### Effect of TMEM45A inactivation on cisplatin-induced UPR activation

**Fig. 6:**
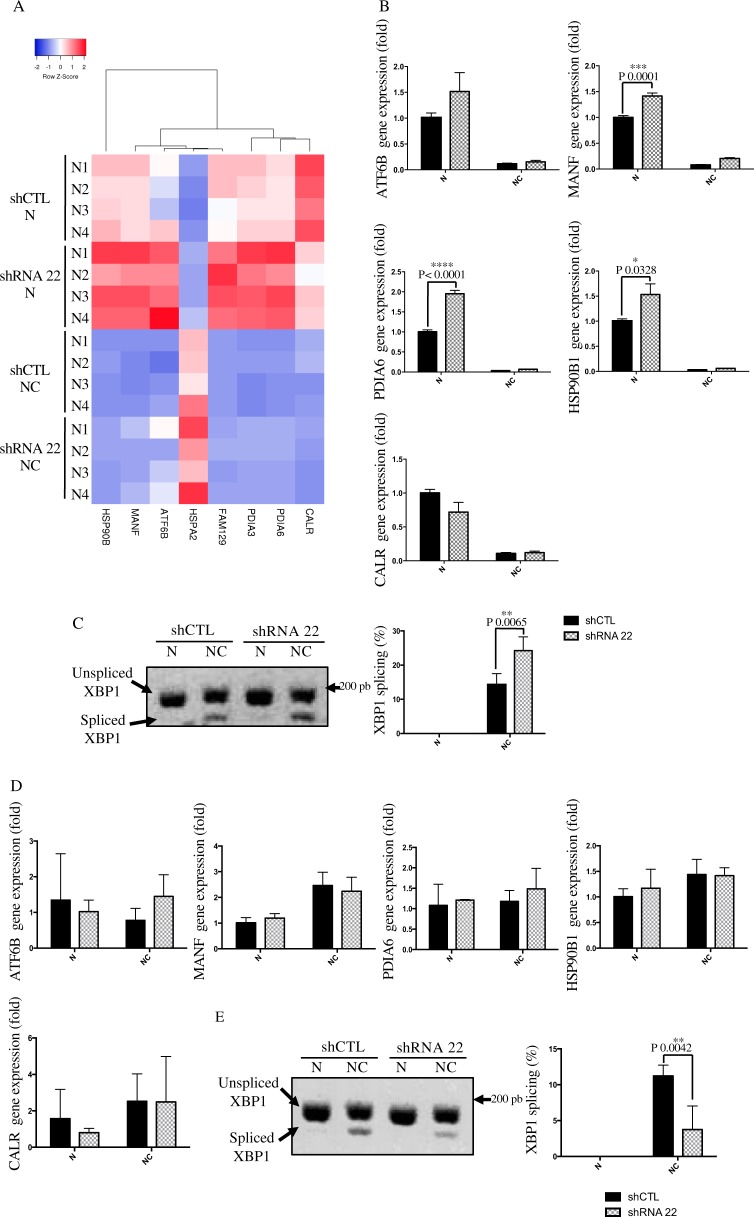
Effect of TMEM45A inactivation on UPR pathway activation. **a**, **b** SQD9 cells were transduced with lentiviral particles expressing shRNA control (shCTL) or shRNA targeting the mRNA of TMEM45A (shRNA 22). The cells were incubated with or without 100 mM of cisplatin in normoxic (N) conditions for 24 h. (A) Genesexpression level was assessed by RNA sequencing. The transcriptome analysis was performed using Babelomics and GSEA after RNA sequencing. Heatmap of differentially expressed genes produced by using the heatmapper^26^ (http://www.heatmapper.ca) showed a potential deregulation of the Unfolded Protein Response activation. Genes have been reordered by the method of average clustering. A dendrogram is shown for the clustering of genes. Genes with relatively high expression are marked in red, genes with relatively low expression are marked in blue. The corresponding values are presented in Supplementary Fig. 4. **b** After mRNA extraction the expression level of several target genes of UPR pathway was assessed by RT-qPCR. **c** SQD9 cells were incubated with or without 20 mM of cisplatin in normoxic conditions for 48 h. Splicing of XBP1 was assessed by PCR and migration on agarose gel. The signal intensity was measured by Image J. Results are expressed as mean ± SD (*n* = 3). **d**, **e** RCC4 cells were transduced with lentiviral particles expressing shRNA control (shCTL) or shRNA targeting the mRNA of TMEM45A (shRNA 22). RCC4 cells were incubated with or without 20 mM of cisplatin in normoxic conditions for 48 h. **d** After mRNA extraction the expression level of several target genes of UPR pathway was assessed by RT-qPCR. **e** Splicing of XBP1 was assessed by PCR and migration on agarose gel. The signal intensity was measured by Image J. Results are expressed as mean ± SD (*n* = 3). N normoxia, NC normoxia + cisplatin. *p < 0.05, **p < 0.01, ***p < 0.001, ****p < 0.0001.

The transcriptome analysis also revealed a potential deregulation of the unfolded protein response (UPR) (Supplementary Fig. 3 and Fig. 6a). Based on the knowledge that TMEM45A is localized to the trans-Golgi apparatus and that protein modification pathways appears to be altered in TMEM45A-knockdown cells (Supplementary Fig. 9A), we decided to further investigate UPR.

The role of UPR activation in regulating cisplatin-induced cell death is controversial. Indeed, some studies showed that UPR activation led to resistance to cell death induced by cisplatin^33,34^, while it has been shown that cisplatin can induce apoptotic signaling via UPR activation, independently of DNA damage in enucleated cells^35^. In fact, a moderate UPR activation is rather anti-apoptotic. However, severe UPR activation leads to apoptosis via the IRE1α (inositol-requiring enzyme-1) and PERK (PRKR-Like Endoplasmic Reticulum Kinase) pathway activation^36^. To study the effect of TMEM45A knockdown on UPR activation, both cell lines were incubated with 20 μM of cisplatin for 48 h. These conditions were chosen since, at earlier time points, no UPR activation could be detected (data not shown).

We first validated RNA-sequencing results using RT-qPCR. For SQD9 cells, we confirmed the increase in the expression of *MANF* (Mesencephalic Astrocyte Derived Neurotrophic Factor), *PDIA6* (Protein Disulfide Isomerase-Associated 6), and *HSP90B1* (Heat Shock Protein 90 kDa Beta Member 1). *ATF6B* (Activating Transcription Factor 6 Beta) and *Calr* (Calreticulin) expression also showed the same tendency, but the statistical significance was not reached (Fig. 6b). On the contrary, these genes did not show any deregulation in RCC4 + pVHL cells (Fig. 6d).

In order to confirm UPR activation, we analyzed the expression of total and phosphorylated forms of IRE1α. It has to be noted that we did not succeed in detecting a band corresponding to the phosphorylated protein in SQD9 cells (data not shown). IRE1α activation was thus analyzed through its unconventional RNA splicing activity of XBP1 (X-box binding protein 1)^37^.

An increase in XBP1 splicing in the presence of cisplatin was observed in both cell lines, confirming the activation of the UPR pathway. More importantly, we observed a significant increase in XBP1 splicing in TMEM45A-knockdown SQD9 cells while this splicing was decreased in TMEM45A-knockdown RCC4 + pVHL cells (Fig. 6c, e). These results suggest that TMEM45A inactivation has opposite effects on the cisplatin-induced UPR activation in the two cell lines.

## Discussion

In this study, we demonstrated that TMEM45A inactivation impacted several functions. Very little is known about this protein. RNA-sequencing analysis evidenced several deregulated pathways associated with golgi apparatus and endoplasmic reticulum. Furthermore, we underlined the complex differential responses upon TMEM45A inactivation according to the cancer cell type.

Among the deregulated pathways, a deregulation of fatty acid biosynthesis (Supplementary Figs. 3 and 9b) was detected. *PTGS2* (Prostaglandin-Endoperoxide Synthase 2), *PTGS1* (Prostaglandin-Endoperoxide Synthase 1), and *ALOX5* (Arachidonate 5-Lipoxygenase) gene products are localized in the endoplasmic reticulum. Metabolic adaptation of cancer cells is a complex process mainly characterized by a switch of mitochondrial oxidative phosphorylation to aerobic glycolysis^38,39^. However, some studies highlighting the role of fatty acid metabolism in cancer proliferation^40–42^. This result means that the absence of TMEM45A in SQD9 cells may induce metabolic changes.

In addition, our results showed an alteration of the protein modification pathway and of UPR indicating that TMEM45A may be involved in post-translation protein modification and cellular trafficking. Indeed, the deregulation of *ICMT* (Isoprenylcysteine Carboxyl Methyltransferase) and *GALNT2* (Polypeptide *N*-Acetylgalactosaminyltransferase 2) genes, which are implicated in post-translational methylation and O-linked glycosylation, and of *INSIG1* (Insulin Induced Gene 1), which mediates protein feedback between endoplasmic reticulum and golgi apparatus supports this hypothesis. These changes possibly lead to the accumulation of unfolded proteins and then to UPR basal activation in TMEM45A- knockdown cells. Furthermore, upon cisplatin exposure, the basal activation induced by TMEM45A inactivation could modify the response to cisplatin. Indeed, in the presence of cisplatin SQD9 cells showed a severe activation of UPR pathway that may lead to cell death whereas RCC4 cells showed a moderate activation of UPR pathway that may confer resistance to cisplatin-induced cell death^33^.

RNA-sequencing analysis also suggested that TMEM45A silencing impacts SQD9 cell responses to cisplatin through the deregulation of DNA damage responses. DNA damage induce the accumulation of p53, leading to the transcription of genes involved in cell cycle checkpoints and in the intrinsic and extrinsic apoptosis pathways^43^. However, p53 is mutated in SQD9 cells. Hence, we investigated upstream events. In SQD9 cells, we observed a decreased in EYA3 expression, which may lead to a lack of dephosphorylation of γH_2_AX and a decrease in RAD51 recruitment to DNA damage foci. These data may explain the increased chemosensitivity observed in SQD9 cells.

The absence of TMEM45A also led to a significant decrease in cell proliferation as already shown in human ovarian cancer cells and in human glioma cells^17,18^, indicating that at least in four different cancer types, cell proliferation is a pathway dependent on TMEM45A expression. For the first time our work provides an explanation for this observation. Indeed, the decrease in proliferation observed in TMEM45A-inactivated cells may be explained by the deregulation of the expression of genes implicated in G2/M transition pathway such as *FANCA* (Fanconi Anemia Complementation Group A) and *ARPP19* (CAMP Regulated Phosphoprotein 19). FANCA is a DNA repair protein, which plays a role in the cell cycle checkpoint and in the maintenance of chromosome stability^44^. ARPP19 inactivates PP2A (Protein Phosphatase 2) in order to keep cyclin B1-CDK1 activity high during M phase^45,46^. The downregulation of these two genes may explain the delay observed in the TMEM45A-inactivated cell proliferation.

This study highlines for the first time the dual role of TMEM45A in drug sensitivity depending of the cancer type. Indeed, TMEM45A knockdown modulated cisplatin-activation of UPR pathway, leading to the resistance of RCC4 + pVHL cells, whereas it increased SQD9 cell sensitivity to cisplatin through the deregulation of cell responses to cisplatin-induced DNA damage. Altogether, these data emphasize the involvement of TMEM45A in tumor aggressiveness and suggest that TMEM45A might be a putative target to develop new chemosensitizing agents at least in some cancer types.

## Supplementary information

Supplementary figure 1

Supplementary figure 2

Supplementary figure 3

Supplementary figure 4

Supplementary figure 5

Supplementary figure 6

Supplementary figure 7

Supplementary figure 8

Supplementary figure 9
