# Supplementary figures and images for: Characterization of the role of TMEM45A in cancer cell sensitivity to cisplatin
Source: Cell Death Dis. 2019 Dec 4;10(12):919. doi: 10.1038/s41419-019-2088-x (PMC6892797; doi:10.1038/s41419-019-2088-x)

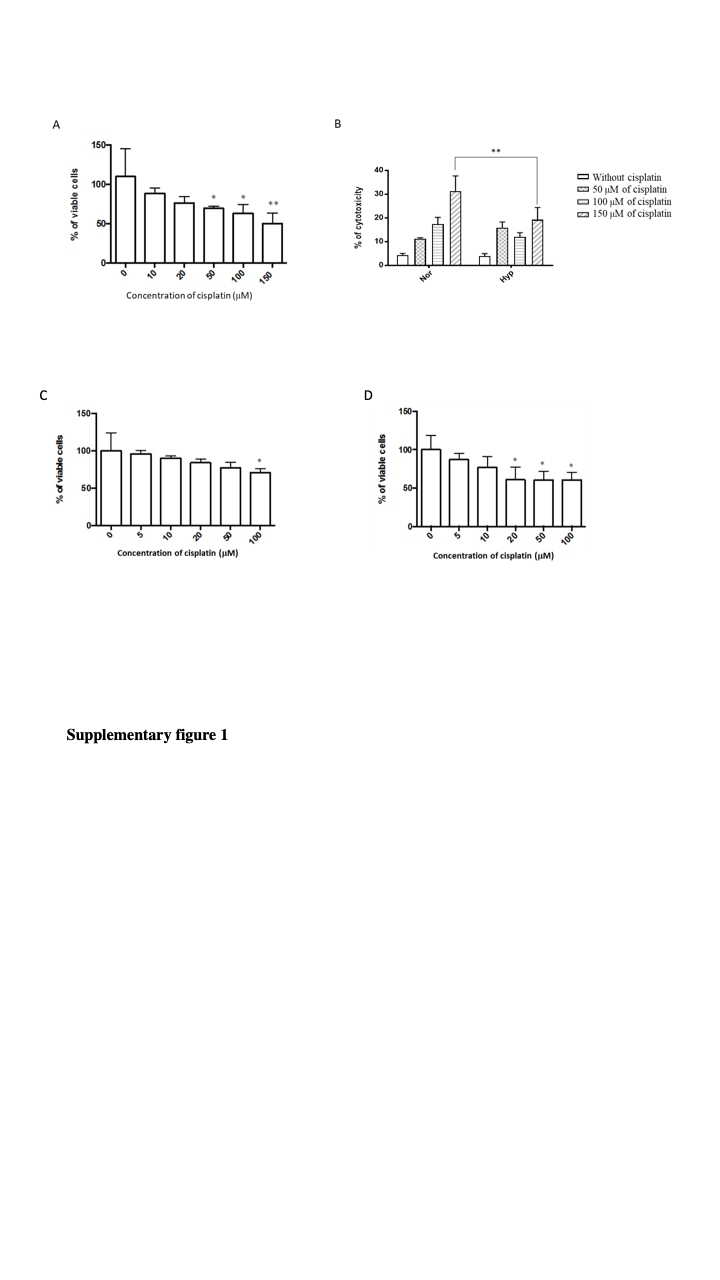

Supplement: Supplementary file 1 — Supplementary figure 1 [file 41419_2019_2088_MOESM1_ESM.png]

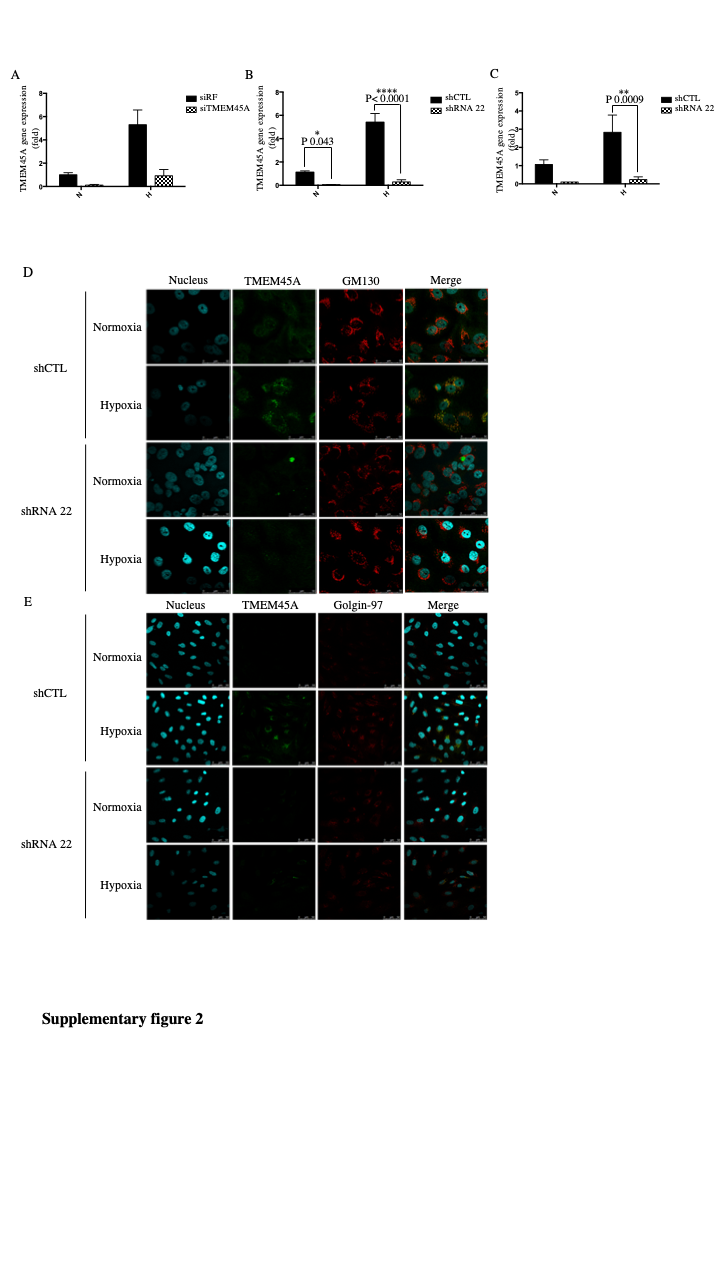

Supplement: Supplementary file 2 — Supplementary figure 2 [file 41419_2019_2088_MOESM2_ESM.png]

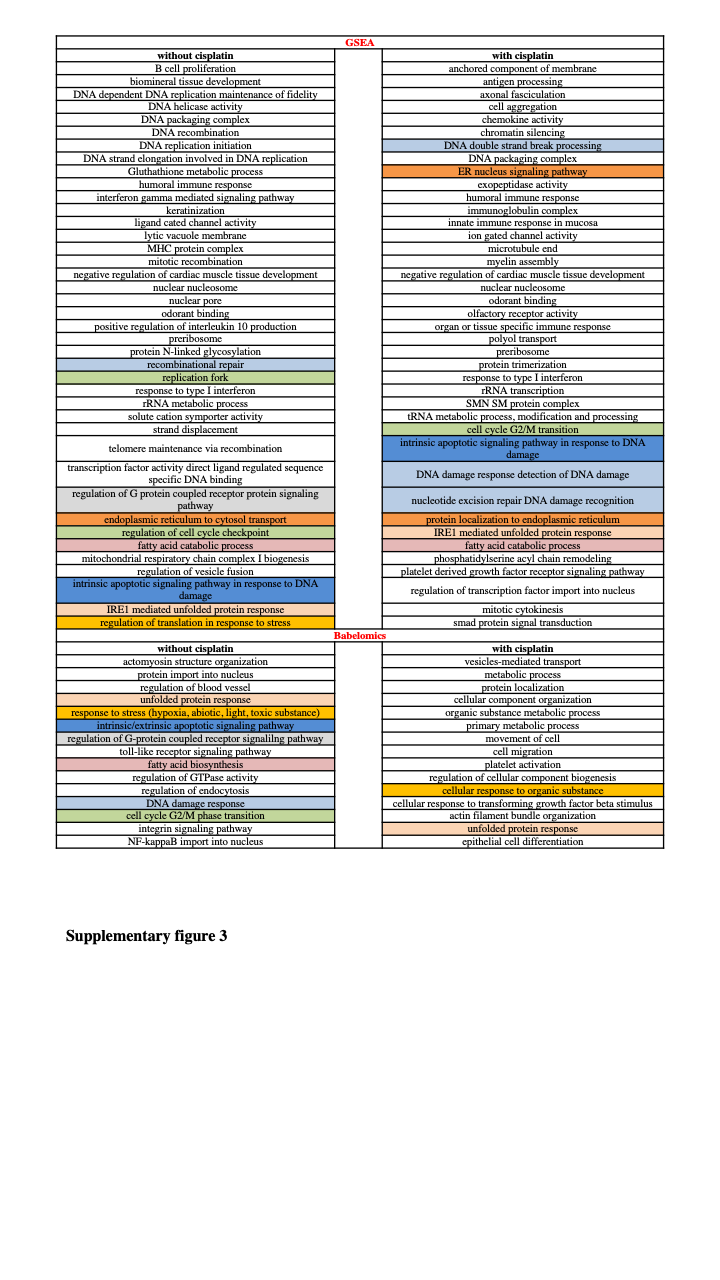

Supplement: Supplementary file 3 — Supplementary figure 3 [file 41419_2019_2088_MOESM3_ESM.png]

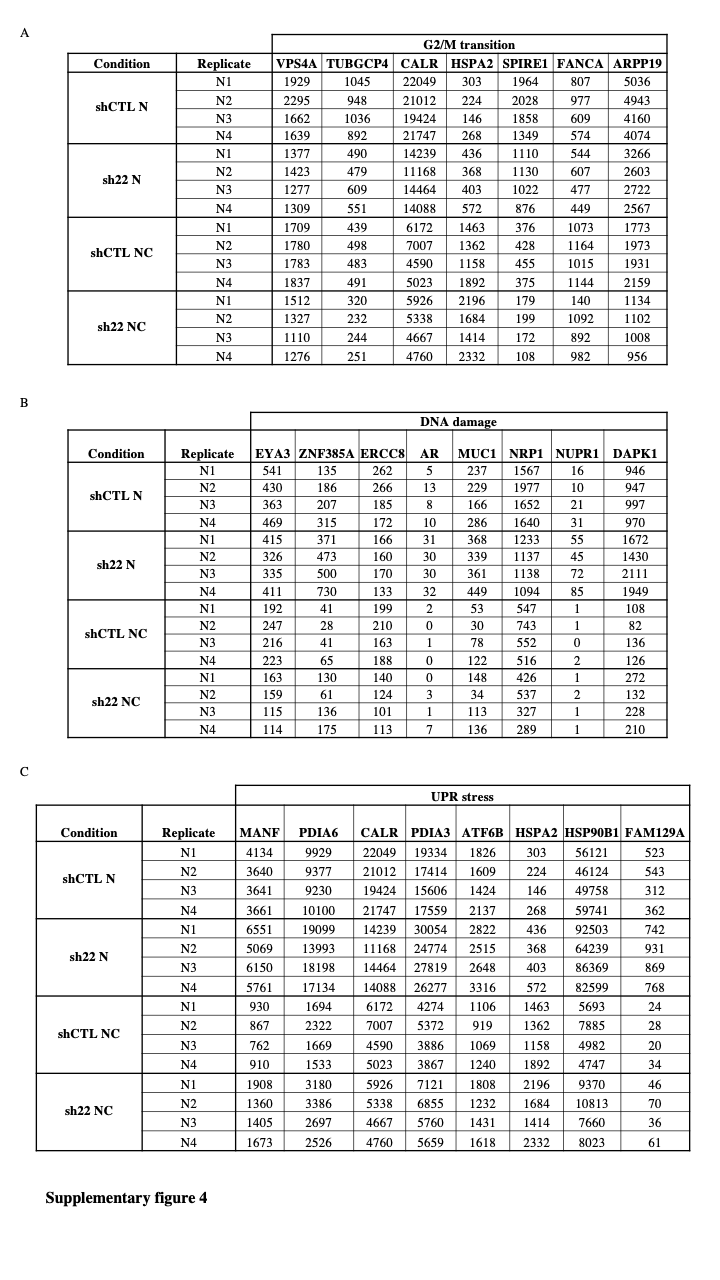

Supplement: Supplementary file 4 — Supplementary figure 4 [file 41419_2019_2088_MOESM4_ESM.png]

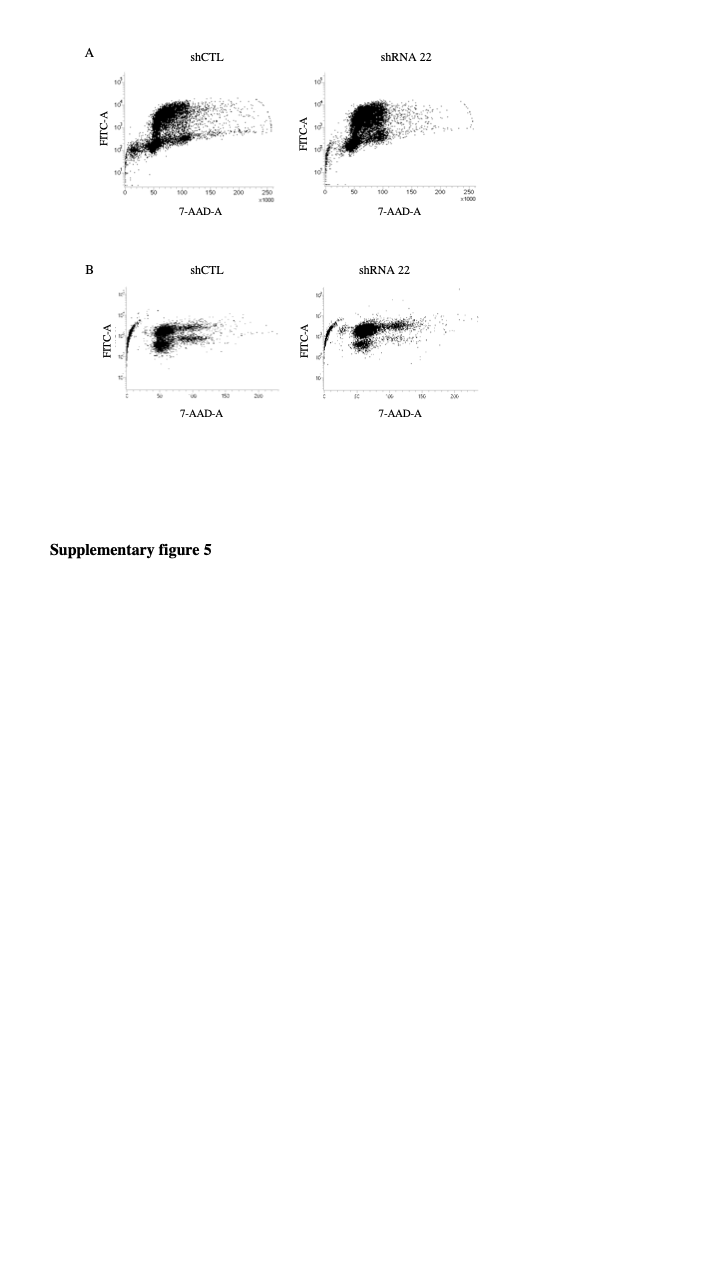

Supplement: Supplementary file 5 — Supplementary figure 5 [file 41419_2019_2088_MOESM5_ESM.png]

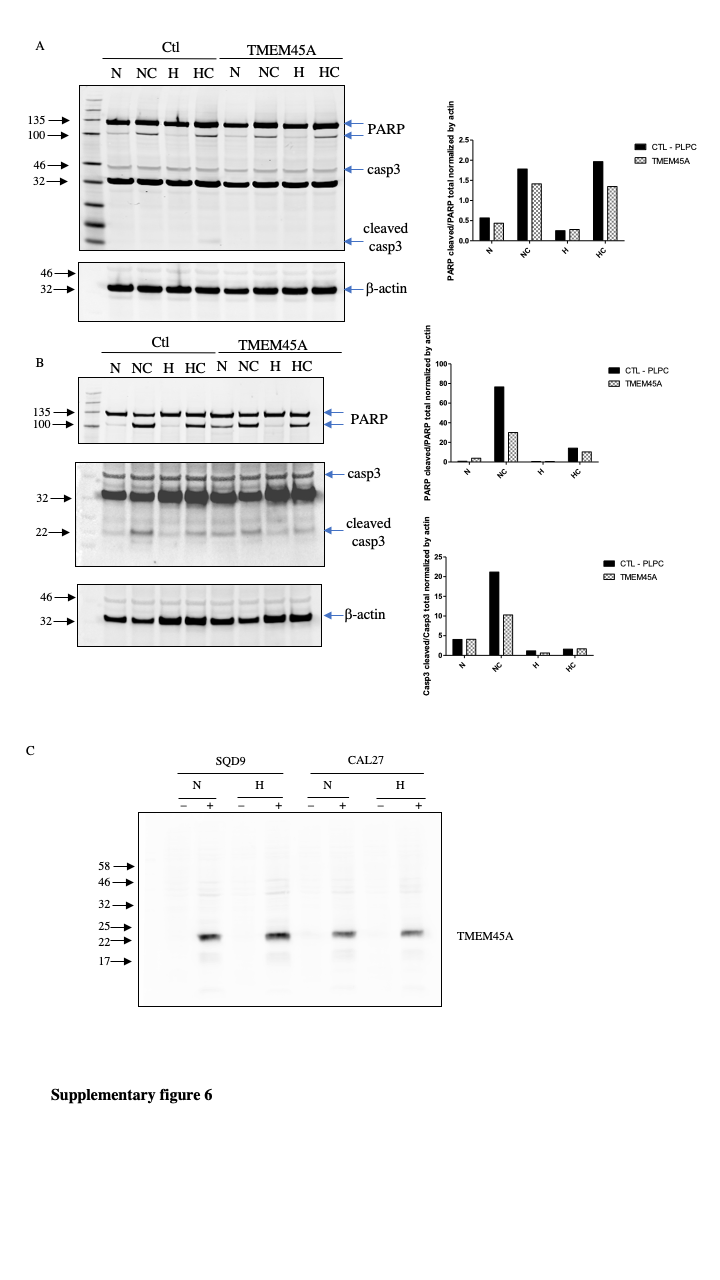

Supplement: Supplementary file 6 — Supplementary figure 6 [file 41419_2019_2088_MOESM6_ESM.png]

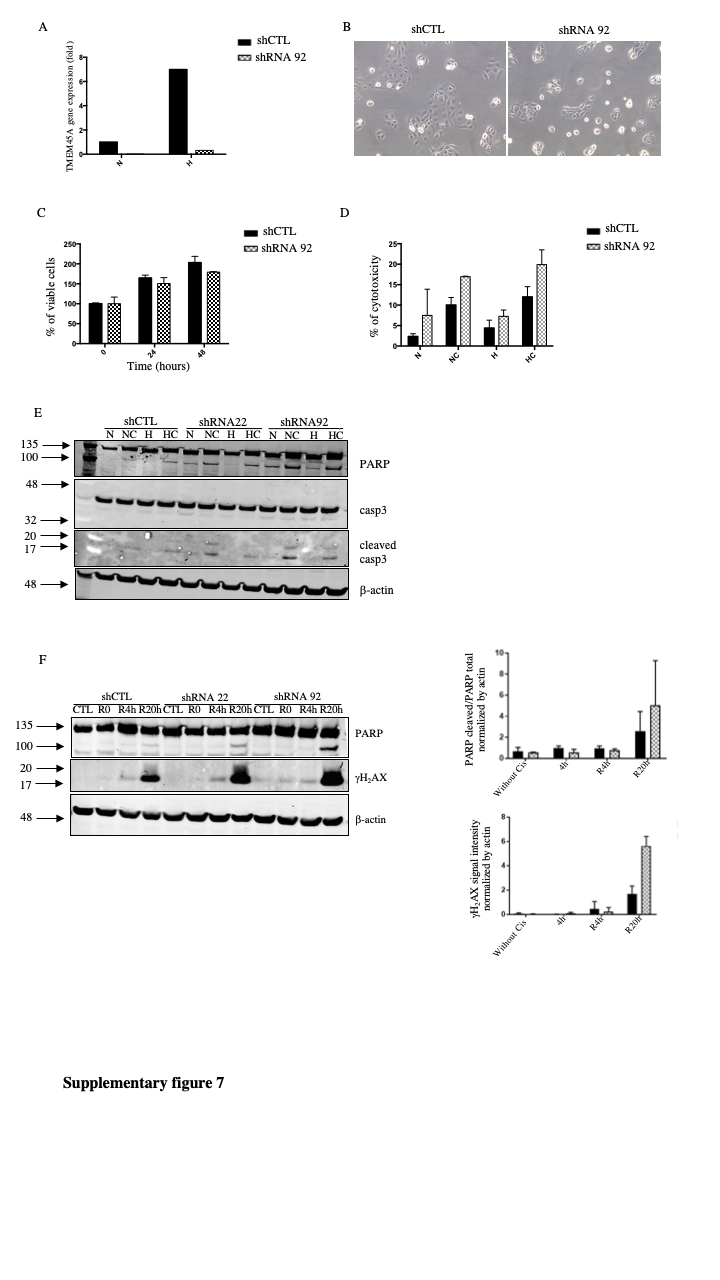

Supplement: Supplementary file 7 — Supplementary figure 7 [file 41419_2019_2088_MOESM7_ESM.png]

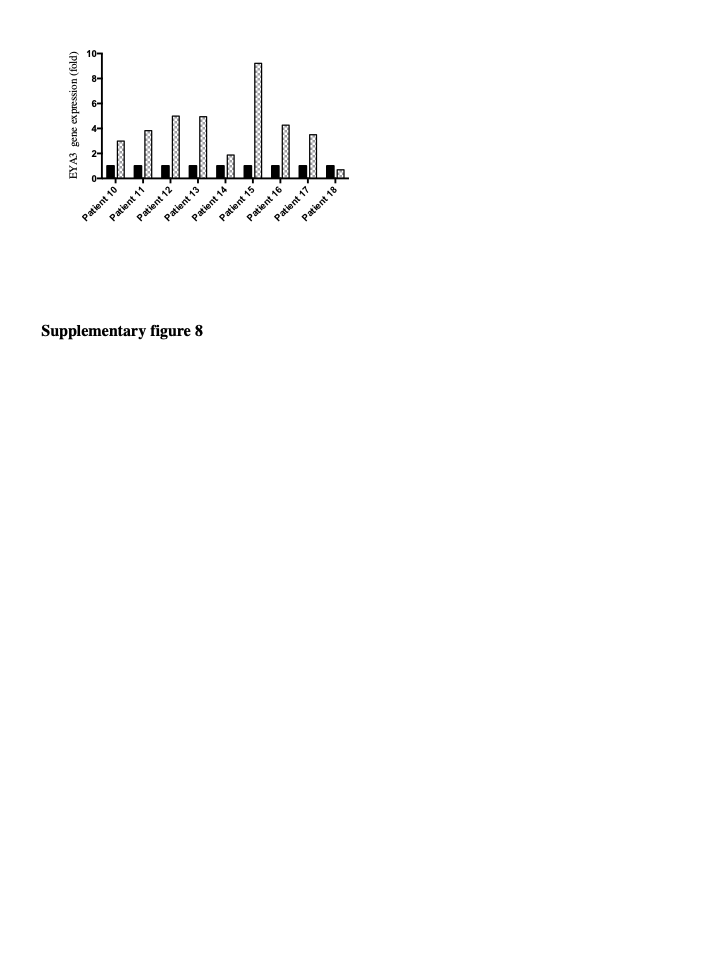

Supplement: Supplementary file 8 — Supplementary figure 8 [file 41419_2019_2088_MOESM8_ESM.png]

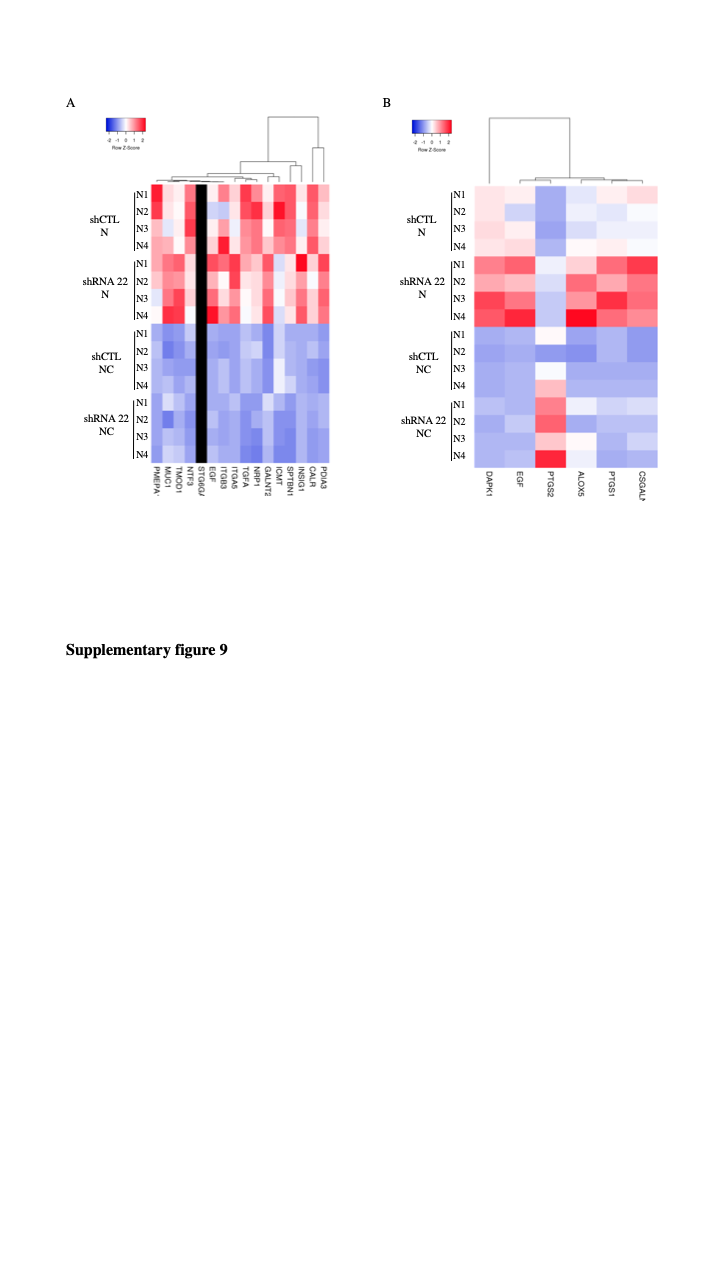

Supplement: Supplementary file 9 — Supplementary figure 9 [file 41419_2019_2088_MOESM9_ESM.png]
